# Exploring public perceptions of solutions to tree diseases in the UK: Implications for policy-makers

**DOI:** 10.1016/j.envsci.2017.06.008

**Published:** 2017-10

**Authors:** Paul Jepson, Irina Arakelyan

**Affiliations:** School of Geography and the Environment, University of Oxford, South Parks Road, Oxford, OX1 3QY, United Kingdom

**Keywords:** Public perceptions, Ash dieback, Chalara, Tree health policy, *Fraxinus excelsior*

## Abstract

Tree diseases are on the increase in many countries and the implications of their appearance can be political, as well as ecological and economic. Preventative policy approaches to tree diseases are difficult to formulate because dispersal pathways for pest and pathogens are numerous, poorly known and likely to be beyond human management control. Genomic techniques could offer the quickest and most predictable approach to developing a disease tolerant native ash.

The population of European Ash (*Fraxinus Excelsi*or) has suffered major losses in the last decade, due to the onset of *Hymenoscyphus fraxineus* (previously called *Chalara Fraxinea*) commonly known in the UK as ash dieback. This study presents evidence on the public acceptability of tree-breed solutions to the spread of *Chalara*, with the main aim to provide science and policy with an up-stream ‘steer’ on the likely public acceptability of different tree breeding solutions. The findings showed that whilst there was a firm anti-GM and ‘*we shouldn’t tamper with nature*’ attitude among UK publics, there was an equally firm and perhaps slightly larger pragmatic attitude that GM (science and technology) should be used if there is a good reason to do so, for example if it can help protect trees from disease and help feed the world. The latter view was significantly stronger among younger age groups (Millennials), those living in urban areas and when the (GM)modified trees were destined for urban and plantation, rather than countryside settings. Overall, our findings suggest that the UK government could consider genomic solutions to tree breeding with more confidence in the future, as large and influential publics appear to be relaxed about the use of genomic techniques to increase tolerance of trees to disease.

## Highlights

• UK publics do not support a ‘let nature take its course’ policy response to the disease *Chalara* affecting UK ash trees.
• Genomic techniques offer a relatively quick and predictable means to develop a native ash tree tolerant to the disease.
• The proportion of respondents who consider this approach acceptable was slightly larger than those who were anti-GM.
• Pragmatic ‘genomics okay where good reason to use’ attitudes were associated with younger and more educated respondents.
• Policy makers could consider genomic solutions to tree breeding with more confidence concerning their public acceptability.

## 1 Introduction

Tree diseases are a global problem and on the increase in many countries due to a number of reasons including globalisation and climate change (Harper et al., 2016): the implications of their appearance and spread can be political, as well as ecological and economic. This is because certain trees species have a place in culture and in the shaping of national and local identities and as a result the visible manifestation of tree diseases can be aligned and amplified with issues and politics beyond the policy domains of silviculture and biodiversity.

This situation arose in the UK following the arrival of the fungal pathogen *Hymenoscyphus fraxineus* (hereafter, *Chalara. The asexual stage of the fungus attacks the bark, twigs and branches of the* European Ash tree *Fraxinus excelsior causing ‘dieback’*. The reproductive stage grows during the summer on fallen leaves of the previous year and the spores are spread by wind. In the UK the Ash is widely known and valued: it has deep cultural, spiritual and literary associations, is used as a timber and fuelwood source (Rackham, 2014) and contributes to the character of iconic landscapes in national parks such as Snowdonia and the Peak District. *Chalara* was first identified in the UK on imported ash saplings in a Buckinghamshire nursery in February 2012. Later in 2012, it was confirmed that *Chalara* was the cause of dieback among a group of ash trees in established woodland sites in the eastern counties of Norfolk and Suffolk (Forestry Commission, 2016) which led to media reporting with headlines suggesting dire consequences for the future of ash trees and commentary that suggested that government had ignored warnings of *Chalara* spreading to the UK. This happened in the context of the UK government announcing a policy to ‘self-off’ public woodlands two years earlier, which was then withdrawn due to the intensity of the public outcry. *Chalara* appeared at a time when influential UK publics were still angered by their government’s apparent disregard for the deep connections between woodlands and cultural identity and as a consequence political leaders came under intense pressure to explain the perceived policy failure associated with *Chalara* and to ‘do something’. The name Ash dieback, rather than *Chalara*, for the disease, caught the UK public’s attention. In this article we use the two names interchangeably depending on context.

Preventative policy approaches to tree diseases are difficult to formulate because dispersal pathways for pest and pathogens are numerous, poorly known and likely to be beyond human management control (e.g. air borne diseases). Given this, and the fact that trees are located (grown) in many different ownerships, cultural and policy contexts, an adaption-based response of developing disease tolerant trees is being considered. Traditional methods involving propagation and crossing of stock from disease tolerant trees is slow because it produces uncertain outcomes and plants need to reach a particular age before features express. Genomic techniques offer the prospect of a more rapid and certain development of disease tolerant trees and the option to enhance other traits that are considered valuable (e.g. strait trunks for timber). Such techniques take two forms: i) genomic screening (termed accelerated breeding) whereby trees are screened at a young age for molecular markers that predict disease tolerance and other features, and ii) genetic modification where genes conferring tolerance are introduced from other species. This latter technique comprises two approaches with policy relevance: Cis-GM where genes from the same species (in this case *Fraxinus*) are introduced, and Trans-GM where genes from a quite different plant species are introduced e.g. common nettle (*Urtica dioica*). In the case of *F.excelsior* research on identifying markers that predict susceptibility to *Chalara* is at an advanced stage: an annotated whole genome assembly of Fraxinus excelsior has recently been published together with transcriptomic and metabolomic work related to ash dieback (Harper et al., 2016, Sollars et al., 2017). The conundrum for policy makers is that whilst genomic science can offer solutions that enable rapid and cost effective breeding, the political controversies surrounding the introduction of agricultural GM technologies in the 1990s inflicted political wounds that have left a legacy of ‘policy fear’ surrounding their adoption.

This paper reports the findings of UK study of public perceptions to different tree-breeding solutions to ash dieback. This study was a component of a wider BBSRC-funded research project that aims to develop new approaches for identifying genes conferring tolerance to *Chalara*. This project is in turn part of a larger programme of research in support of the UK Plant Biosecurity Strategy for Great Britain (DEFRA, 2014), which was a response to the aforementioned public concern over ash dieback. One important insight from the GM controversy in the 1990s was that societal acceptability of new technologies requires an open public dialogue during the development of the technology (Macnaghten et al., 2015). The goal of this study is to provide science and policy with an ‘upstream steer’ (cf. Kearnes et al., 2006) on the public acceptability of different tree-breeding solutions and in particular those involving genomic techniques. Put another way, scientific research to deliver policy solutions can involve significant cost over the long term. Policy makers and scientists need evidence on the public acceptability of the policy options available in order to orientate research and/or design public awareness campaigns to increase the acceptability of policy. Furthermore, policy needs data on the acceptability of solutions among different publics so they can evaluate the degree of support for different positions in a public debate.

To date research on public perceptions of tree diseases and potential solutions is limited. A large-scale survey of the public acceptability of planting transgenic American chestnut (*Castanea dentata*) was conducted in the US in 2015 (Needham et al., 2015). Preliminary findings showed that support for GM is influenced by environmental values, perceptions of risk, and demographic characteristics. The findings reported here and in Jepson and Arakelyan (2017) add to this knowledge base and the ideal of creating trans-national tree health policy that is realistic, cost-effective and attuned to culture and public attitudes.

Our larger study had three sequential components: 1) a questionnaire survey of ‘interested publics’, meaning those actively engaged with countryside-related practices and associated with public groups active in nature-related public policy discourses; 2) focus group discussions with experts from different sectors engaged with tree health issues and who advise government on policy, and 3) a questionnaire survey of the ‘general’ UK public administered by a commercial survey company. The findings of components 1 and 2 are reported in Jepson and Arakelyan (2017) and Jepson and Arakelyan (2016) respectively. This paper reports the findings of component 3 and compares these with those of the first two. As such it concludes this mixed-method study and our assessment of the extent to which genomic tree breeding techniques might garner public support or opposition.

## 2 Methods

### 2.1 Study design

We adopted an iterative study design that: 1) surveyed the attitudes of informed British publics (N = 1152) likely to be interested in the fate of ash trees and engage discursively and/or politically with tree health issues conducted in July-September 2015 (the rationale for this is provided in Jepson and Arakelyan (2017): 2) discussed tree health policy, the merits of tree-breeding solutions and preliminary findings of the above survey in three focus group discussions with i) media professionals, ii) foresters and forestry industry representatives including nursery and woodland managers and iii) representatives of the government and major trusts involved in forest research, conducted in November 2015 and, 3) conducted a representative survey of the UK public administered by YouGov in March 2016 that incorporated insights from the first two components whilst maintaining comparability with the first questionnaire survey.

An account of the *Chalara* outbreak in the UK and the methods used in the first survey (Phase 1) are presented in Jepson and Arakelyan (2017). In brief, we developed a questionnaire instrument that measured acceptability of seven tree-breeding solutions to ash dieback and a “no action” option, and administered this at three countryside events that market to distinct publics: namely the Country Landowners Association Game Fair (rural land owners, workers and sports); the British Birdwatching Fair (naturalists); and the Royal Horticultural Society (RHS) Wisley Flower show (gardeners). We adopted a quota and surveyed 400 people at each event based on Dillman et al. (2014) calculation that a sample size of 384 respondents can be projected to a population of ≥1,000,000 people with a confidence interval of 95%.

The questionnaire from Phase 1 was adapted for on-line administration to a general public and to incorporate insights from the results of the first survey. The key changes made were: a) a Cis-GM and a Trans-GM solution were combined into a single GM option and respondents were asked to check the three options most acceptable to them, from the list of 7 options (Box 1) (in the first survey respondents were asked to rank the three most acceptable options and check the two least acceptable options); b) the online questionnaire had a stronger focus on 3 specific tree breeding solutions, including the use of GM-techniques, planting non-native disease tolerant ash trees, and planting hybrids of native ash tree with non-native ash trees. This was because these options are currently seen by experts as the most feasible options to deal with ash dieback. In particular, respondents were asked how acceptable or otherwise they would find any of these options in urban areas, forestry plantations and natural woodlands. In addition, a new question on respondents’ living location was included because the Phase 1 survey findings suggested that acceptability of different breeding solutions might be influenced by whether the respondent is an urban or rural resident.

Box 1 **Question set of solution options included in YouGov on-line survey. Respondents were asked to check the three options most acceptable to them, with approximate timescales given for the implementation of each option on the list.**

1. Planting different native (broadleaf) tree species (e.g. oak) to replace ash trees (5 years)
2. Planting non-native species of ash trees (e.g. Manchurian or Asian ash trees) that are more tolerant to ash dieback (5 years)
3. Breeding native tolerant ash trees using traditional techniques (25+ years)
4. Cross-breeding native ash tree with non-native ash tree to create disease tolerant hybrids (20+ years)
5. Using accelerated (genomic) breeding to breed native tolerant ash trees (15+ years)
6. Genetically modifying native ash trees to enhance disease tolerance (5–10 years)
7. No action - letting nature take its course (75+ years)

Alt-text: Box 1

The questionnaire was reviewed by experts from YouGov Plc and adjustments made to improve clarity and ease of completion (See annex 1 for survey). It was sent by email on 15 April 2016 to a sample selected at random from the base sample of 800,000+ UK adults who have agreed to take part in YouGov surveys. The profile of the sample is derived from census data or, if not available, from other industry accepted data.

The figures have been weighted and are representative of all GB adults (aged 18+).

### 2.2 Data analysis

Data was analysed using Statistical Package for Social Sciences (SPSS) 22.0 software. Three complimentary analytical approaches were deployed: i) basic descriptive statistics, ii) chi-square tests to investigate the relationship between variables, iii) logistic regression models to investigate various factors that affected the likelihood of respondent’s selection of a particular option. The main hypotheses were based on the findings from the previous 2 phases of the project (survey of interested publics, and focus groups with experts), as well as on research findings from previous studies.

#### 2.2.1 Empirical models

A characterisation was done using contingency tables (cross tabulation) to compare the proportion of respondents who selected or did not select a particular option to deal with ash dieback. Selection of a particular option was quantified using a binary variable (selection of an option = 1, non-selection = 0). Chi-square tests were carried out to assess relationships between selection and socio-economic variables. A standard logistic regression model (logit model) was used in a binary choice (selection versus non-selection of option) of outcomes. The model provides empirical estimates of how change in the socio-economic and exogenous variables influences the probability of selection and assesses the effectiveness of the selection of various options to deal with ash dieback (Nkonya et al., 1997).

A logistic function including odds ratios was used to derive coefficients of explanatory variables likely to influence respondents’ attitudes to the selection of various options. Selection is a dichotomous variable (selector = 1/non-selector = 0) and 4 out of 5 independent variables are also categorical.

7 different models were used for the study, looking at the impact of the independent variables on 7 different options to deal with ash dieback.

The binary logistic model used in the study is specified as follows (adapted after Quddus, 2012):

$$P=p\left(Y=\frac{1}{X}\right)=\frac{e^{\beta 0+\sum_{i=1}^{10}\beta iXi}}{1+e^{\beta 0+\sum_{i=1}^{10}\beta iXi}}$$

and,

$$1-P=p\left(Y-\frac{0}{X}\right)=\frac{1}{1+e^{\beta 0+\sum_{i=1}^{10}\beta iXi}}$$

where Yi (the dependent variable) is the level of technology adoption (i.e. adoption of new options/technologies to deal with ash dieback) (1 = adopters, 0 = non-adopters);

A transformation of P known as the logit transformation and is defined as:

$LogitP=\log\left[\frac{P}{1-P}\right]=\beta o+\overset{10}{\underset{i=1}{\sum }}\beta iXi$

#### 2.2.2 Dependent and explanatory variables

We focus on analysing the determinants of the likelihood of respondents’ selection of 7 different options to deal with ash dieback (dependent variables, as listed in Box 1).

Based on the data availability we have selected a range of respondents’ characteristics that are hypothesized to influence their choice of options. These include: respondent’s gender, age, level of education, as well as respondent’s location (urban or rural), and the proposed planting location of improved ash trees or substitute trees. The main hypotheses for each explanatory variable are presented in Table 1.

**Table 1:** Explanatory variables and the summary hypotheses for Phase 2 (survey of general UK population).

Variable	Hypothesis
X1: GENDER (takes the value of 1 if male and 0 otherwise)	Gender does not play a significant role in attitude to tree breeding solutions.
X2: AGE (continuous)	Older respondents are more likely to be against GM solutions to ash dieback, and more in favour of natural breeding solutions.
X3: EDUCATION (takes the value of 1 if a degree, and 0 otherwise)	Respondents with a degree are more likely to be in favour of higher degree intervention approaches, such as GM.
X4: RESPONDENT LOCATION (takes the value of 1 if rural, and 0 otherwise)	Rural residents will be more conservative in their choice of options preferring the low degree of intervention, natural breeding options, while urban residents will be less supportive of GM and planting non-native ash options in urban settings.
X5: PLANTING LOCATION (takes the value of 1 if natural woodlands, and 0 otherwise)	Respondents will be more accepting of GM, non-native ash, and hybrid trees options in commercial forestry plantations and urban areas, and less accepting of these options in natural woodlands and wider countryside.

## 3 Results

### 3.1 Survey responses

A total of 2036 completed surveys were returned. The respondent population was broadly representative of the general English population in terms of gender and education, which support statistically valid comparison (Table 2):

**Table 2:** Breakdown of YouGov return survey population by category (UK adults, N = 2036).

Gender	Male	977 (48%)
	Female	1059 (52%)

Age	18–24	244 (12.0%)
	25–34	299 (14.7%)
	34–44	373 (18.3%)
	45–54	407 (20.0%)
	55+	713 (35.0%)

Education	University degree	684 (33.6%)

Location	Urban	1670 (82%)
	Town and Fringe	209 (10.3%)
	Rural	155 (7.6%)

### 3.2 Respondents choice of potential solutions to ash dieback

In our sample of 2036 adults the *do nothing* option received little support: only 14.4% (N = 294) of respondents selected it as one of their three most preferred options. Breeding native ash using conventional means was the most preferred option selected by 40.4% (N = 822) followed by planting different native tree species (31.6%, N = 643), accelerated (genomic) breeding (30.3%, N = 618) and genetically modifying ash trees (27.3%, N = 555). The survey revealed limited acceptability for planting involving non-native trees: the option of replacement with a non-native ash species appeared in the top three choices of just 17.3% (N = 353) of respondents and cross-breeding native and non-native ash trees in 17.6% (N = 357) (Table 3).

**Table 3:** The preferred tree breeding solutions to Ash dieback, in a descending order, as an overall proportion of respondents (total number of respondents 2036) (number of respondents per individual response reported in brackets). Q. Please select your three most preferred (top) options from the list (of potential solutions to deal with ash dieback), taking into account the approximate timescale necessary to implement each option.

Course of action to deal with ash dieback	Total
Breed native tolerant ash	**40.4% (N = 822)**
Plant different native species	**31.6% (N** = **643)**
Use accelerated breeding	**30.3% (N** = **618)**
Use GM-techniques, including cis-genetics and trans-genetics	27.3% (N = 555)
Cross native ash X non-native ash	17.6% (N = 357)
Plant non-native tolerant ash	17.3% (N = 353)
No action	14.4% (N = 294)

Chi-square test results showed a significant association (p < 0.001 for all) between respondent’s age, and between their most preferred solutions to deal with *Chalara*, namely, planting different native tree species, use of GM-techniques to develop diseases-tolerant ash trees; cross-breeding native ash with non-native ash; and natural breeding (Table 4).

**Table 4:** Preferences of respondents to various solutions to deal with *Chalara*, by age groups.

Course of action to deal with ash dieback	18–24	25–34	35–44	45–54	55+	χ^2^
Plant different native tree species	23.4% (57)	23.3%(70)	29.0% (108)	37.7%(153)	35.8% (255)	31.164***
Use genetic modification (GM) techniques to develop disease-tolerant ash trees (5–10 years)	38.4% (94)	22.7% (68)	19.3% (72)	26.6% (108)	30.0% (214)	33.008***
Cross-breed native ash with the Asian ash (i.e. non-native) to create a disease-tolerant hybrids (20+ years)	22.4% (55)	14.7% (44)	24.1% (90)	16.5% (67)	14.3% (102)	22.293***
Natural breeding	33.2% (81)	31.7% (95)	37.0% (138)	43.7% (178)	46.5% (331)	29.391***

Note: *** significant at 1% level.

Results also showed that younger respondents were more likely to be in favour of methods involving a higher degree of scientific intervention such as the GM options, and older respondents were more likely to be in favour of traditional breeding techniques, and planting different native tree species. An option of cross-breeding native ash trees with non-native (Asian) ash trees received a higher support amongst younger age groups.

We further found that higher levels of education were associated with a higher level of acceptance of GM solutions to tree breeding. In addition, we found that female respondents were likely to be less accepting of GM techniques to deal with *Chalara* than were males (Table 5).

**Table 5:** Proportion of respondents who ranked GM-techniques as one of their top three options, by gender and level of education.

	Level of education (N = 533)		Gender (N = 555)	
	Degree	No degree	Male	Female
	65.7% (350)	34.3% (183)	33.6% (328)	21.5% (227)
**χ^2^**	84.068***		37.597***	

Note: *** significant at 1% level.

Respondents were further asked about the possible influence of a number of events, such as the Dutch elm disease in the 1980s, or ash dieback outbreak in 2012, on their attitude to tree diseases and potential solutions to deal with *Chalara*. We found that older respondents in particular (55+) reported having been very influenced by these two events (p < 0.001) when thinking of tree diseases and solutions to *Chalara*.

### 3.3 The influence of respondents’ location on their attitude to solutions to Chalara

Results showed that respondents’ attitude to four options, namely i) planting non-native ash trees, ii) planting different native species, iii) breeding native ash and iv) ‘no action’ was strongly associated with whether they lived in an urban or rural areas. For example, respondents living in urban areas were more likely to accept planting non-native tolerant ash than respondents living in rural areas. In contrast, respondents living in rural areas were more in favour of planting different native tree species and breeding native tolerant ash than urban residents. Importantly, chi-square test results showed no association between respondents’ location, and attitudes to GM solutions to deal with *Chalara* (Table 6).

**Table 6:** Most preferred options to deal with *Chalara*, by urban/rural location (N = 2034) (Phase 3) (number of respondents reported in brackets, chi-square test results shown for options which showed a significant association with respondents’ location).

Course of action	Respondent’s location		
	Urban	Town and Fringe	Rural
Plant non-native tolerant ash* (**χ^2^ 8.592, p <** **.05)**	**18% (300)**	**19% (39)**	**9% (14)**
Plant different native species*(**χ^2^ 8.465, p <** **.05)**	**30% (505)**	**38% (79)**	**37% (58)**
Use GM-techniques	28% (464)	22% (47)	29% (45)
Use accelerated breeding	29% (491)	31% (66)	39% (61)
Cross-breed native ash with non-native ash	18% (305)	16% (34)	11% (17)
Breed native tolerant ash* (**χ^2^ 10.871, p <** **.05)**	**40% (667)**	**36% (75)**	**51% (79)**
No action** (**χ^2^ 13.147, p < 0.005**)	**14% (233)**	**16% (33)**	**17% (26)**

Note: ** significant at 5% level, * significant at 10% level.

When asked to state preferences on four options (2 involving non-native ash, and 2 involving GM technologies) in three different settings (urban areas, forestry plantations and natural woodlands) we found that all four options had a higher acceptance level if implemented in urban areas and forestry plantations as opposed to natural woodlands (Table 7). We found that the youngest age group (18–24 year olds) showed the highest level of acceptance of GM solutions in all three settings, while the oldest age groups (55+) showed the lowest level of acceptance. Again, female respondents preferred less interventionist approaches (such as natural breeding, and planting of other native trees) compared with males (p < 0.001 for all, Table 8).

**Table 7:** Proportion of respondents opting for a given course of action to deal with *Chalara* to be carried out in three different settings − urban areas, forestry plantations, and natural woodlands (number of respondents is given in brackets) (N = 2036). Q. How acceptable are any of these options in the following places?

Course of action	Urban parks/roadsides/gardens		Forestry plantations		Natural woodlands and wider countryside	
	Unacceptable	Acceptable	Unacceptable	Acceptable	Unacceptable	Acceptable
Planting disease tolerant non-native ash	13% (261)	**42% (846)**	15% (313)	**40% (822)**	23% (462)	33% (674)
Cross-breeding native ash with Asian ash	11% (220)	**43% (884)**	12% (246)	**42% (847)**	18% (375)	35% (718)
Planting cis-GM ash trees	16% (322)	**38% (768)**	16% (336)	**37% (751)**	20% (408)	32% (654)
Planting trans-GM ash trees	16% (330)	**35% (713)**	17% (343)	**34% (683)**	21% (420)	30% (604)

**Table 8:** Acceptability of solutions to *Chalara* in various settings, by gender, acceptable and highly acceptable (N = 2036).

Option	Urban parks/roadsides/gardens		Forestry plantations		Natural woodlands and wider countryside	
	Females	Males	Females	Males	Females	Males
Planting disease tolerant non-native ash	37.6% (398)	45.9% (448)	36.1% (383)	44.9% (439)	29.8% (315)	36.7%(359)
**χ^2^**	24.704***		24.281***		17.961***	
Cross-breeding native ash with Asian ash	39.0% (413)	48.2% (471)	36.7% (389)	46.8% (457)	32.1% (340)	38.7% (378)
**χ^2^**	25.598***		27.587***		21.189***	
Planting cis-GM ash trees	30.0% (318)	46.1% (451)	29.9% (316)	44.6% (436)	26.8% (284)	37.9% (370)
**χ^2^**	57.257***		49.214***		31.366***	
Planting trans-GM ash trees	27.0% (286)	43.7% (427)	25.8% (273)	42.1% (411)	22.2% (235)	37.7% (369)
**χ^2^**	64.072***		61.865***		62.867***	

Note: ***significant at 1% level.

### 3.4 Results of the logistic regression models

Seven logistical regression models were run to explore the factors that influenced respondents’ choice of seven different options to deal with ash dieback. Out of seven models, only four (planting different native tree species; planting non-native species of ash; breeding native tolerant ash, and the use of GM-techniques) were statistically significant, with the remaining three models (exploring the influence of independent variables on respondent’s choice of cross-breeding native ash with non-native ash; use of accelerated/genomic breeding, and no action) showing no significant relationship between the independent variables and the selected option.

The results of the four models (Table 9) provide insights into the significant explanatory variables, which acted as the main driving forces behind respondents’ likelihood of selecting one option to deal with ash dieback over the other. A test of full models against constant only models was statistically significant for all 4 models, showing a strong explanatory power (χ2 = 55.886, p < 0.000 for Model 1; χ2 = 114.520, p < 0.000 for Model 2; χ2 = 148.750, p < 0.000 for Model 3; and χ2 = 56.510, p < 0.000 for Model 4).

**Table 9:** Results of logistic regression analysis predicting likelihood of the selection of various options deal with ash dieback (Models 1–4).

Characteristic	Likelihood of selecting various options to deal with ash dieback (Models 1–4)											
	Model 1: Planting different native tree species (N = 2036)			Model 2: Planting non-native species of ash trees (N = 2036)			Model 3: Breeding native tolerant ash (N = 2036)			Model 4: Use GM-techniques to enhance disease tolerance (N = 2036)		
	Coefficient	S.E.	Exp(B)	Coefficient	S.E.	Exp(B)	Coefficient	S.E.	Exp(B)	Coefficient	S.E.	Exp(B)
Constant	5.962	1.430	0.187	4.416	1.127	1.089	4.519	1.756	1.006	1.437	0.707	0.812
Gender (1 if female, 0 otherwise)	1.625	0.492	0.270	−0.751	0.137	0.025	0.511	0.119	0.251	−0.726***	0.292	2.003
Age (cont)	0.960***	0.319	2.612	0.226	0.348	0.879	.289***	0.005	7.026	−0.603***	0.123	4.202
Education (head) (1 if a degree, 0 otherwise)	0.133	0.258	1.195	−0.467	0.367	1.003	0.184	0.286	0.991	0.314**	0.133	2.460
Respondent location (1 if rural, 0 otherwise)	0.390***	0.003	3.749	−0.386**	0.098	2.732	.395***	0.007	3.694	−0.050	0.053	0.991
Planting location (1 if natural woodlands, 0 otherwise)	0.903	0.222	0.562	−0.395***	0.582	4.230	0.792***	0.145	2.557	−0.904***	0.213	4.801
Model χ2	55.886***			114.520***			148.750***			56.510***		
H-L test	0.863			0.919			0.778			0.748		
Nagelkerke pseudo-*R^2^*	0.437			0.342			0.373			0.560		

Note: *** significant at 1% level, ** significant at 5% level.

Nagelkerke’s pseudo-R2 values for all four models indicated a strong relationship between prediction and grouping. Prediction success overall ranged between 80.9% and 89.4%.

The results for Model 1 (planting different native tree species) demonstrated that the age of a respondent, and their location (urban or rural) are the factors that significantly affected the likelihood of selection of this option. Exp (B) value indicates that older respondents were 2.6 times more likely to select this option, as opposed to younger respondents. Further, respondents living in rural areas were 3.7 times more likely to select this option than respondents living in urban areas.

In Model 2 (planting non-native species of ash trees) only 2 out of 5 predictor variables – respondent’s location, and planting location – were statistically significant. Results showed that respondents living in rural locations were nearly 3 times less likely to support planting non-native species of ash trees, than respondents living in urban locations. Further, all respondents were 4 times less likely to support planting non-native ash trees natural woodlands, than in urban areas and forestry plantations.

Model 3 (breeding native tolerant ash) results showed that age, respondent location and planting location all played a significant role in the selection of this option, with regression coefficients being large and significant at p < 0.000. Older respondents were 7 times more likely to support this option. Further, respondents from rural locations were nearly 4 times more likely to support this option, and overall, respondents were 2.5 times more likely to favour breeding native tolerant ash in natural woodlands than in forestry plantations, or urban settings.

Finally, Model 4 (use of GM-techniques to enhance disease tolerance) results showed that several factors, including gender, age, education and planting location, played a significant role influencing the selection of this option. While for Models 1–3 gender did not play a significant role, Model 4 results showed that female respondents were 2 times less likely to support the use of GM-technique than males. The likelihood of selecting this option decreased 4 times with increasing age, i.e. older respondents were 4 times less likely to support the use of GM-techniques. Unlike in Models 1–3, education did play a role here, and respondents with a university degree were 2.4 times more likely to be in favour of GM-techniques than respondents without a degree. Finally, all respondents were 4 times less likely to support the use of GM-techniques in natural woodlands, than in urban settings or forestry plantations.

### 3.5 Level of acceptability of GM trees

When asked whether they consider it more or less acceptable to genetically modify UK native trees compared with agricultural food crops the majority of respondents (43%) made no distinction between the two considering them equally acceptable/unacceptable. Of the remainder, 20% of respondents thought it was less acceptable to genetically modify trees and 12% found genetic modification of trees more acceptable than genetically modifying agricultural crops (Table 10).

**Table 10:** Reasons for preferring GM trees over GM crops, or otherwise (N = 2036). Q. Do you generally find it more or less acceptable to genetically modify UK native trees, compared with genetically modifying agricultural food crops?

Level of acceptability	Percent	Main reason	Second main reason
More acceptable	12%	Don’t eat trees/not part of a food chain (46%)	Good for trees/prevents diseases/protects trees/stops them dying out (27%)
About the same	43%	Good for the trees/prevents diseases/protects trees/keeps them healthy/stops them dying out (20%)	Both are unacceptable (10%)
Less acceptable	20%	Should not interfere with nature (41%)	Disagree with any genetic modification (13%)

The data revealed a significant association (p < 0.001) between respondents’ attitude to GM food and crops and their attitude to the use GM techniques for tree breeding. Respondents who were more accepting of GM trees as opposed to GM crops, were more likely to be in favour of GM solutions to *Chalara* in general, but also more in favour of GM ash trees planted in urban settings, forestry plantations and natural woodlands.

Respondents were invited to explain why they held a particular view on applying GM techniques to trees compared with crops: 63% 66% (N = 1339) of respondents contributed a view and this question generated clear insight into different basis of attitudes. Of those providing a comment 14.3% (192) saw no difference between crops and trees (*both are plants: same difference*) and this figure increases to 27.8% (N = 372) if those stating they were simply against GM are added. However the majority of respondents did see a difference between trees and crops. The three most prominent views were those coded as ‘*help to protect/save trees*’ (18.4% N = 246); ‘*shouldn’t tamper with nature*’ (12.2% N = 163) and ‘*We don’t eat plants*” (9% N = 121). Other significant viewpoints were ‘*GM is the direction of (scientific) progress’ (4.2% N = 56);* GM is acceptable if there is ‘*a good reason*’ for it (3% N = 41), and we ‘*need to feed growing population*’ (2.5% N = 34).

Simple word frequencies of respondent comments revealed that those who felt it was wrong to apply genetic modification techniques to trees expressed this view using the words ‘interfering’, ‘messing’, ‘tampering’ and ‘meddling’ with nature. In contrast, those who saw genetic modification techniques as ‘helpful’ for trees expressed a wider range of views relating to protecting and saving native trees through making them more resistant to pests and diseases.

### 3.6 Public attitudes to science

**Fig. 1:**
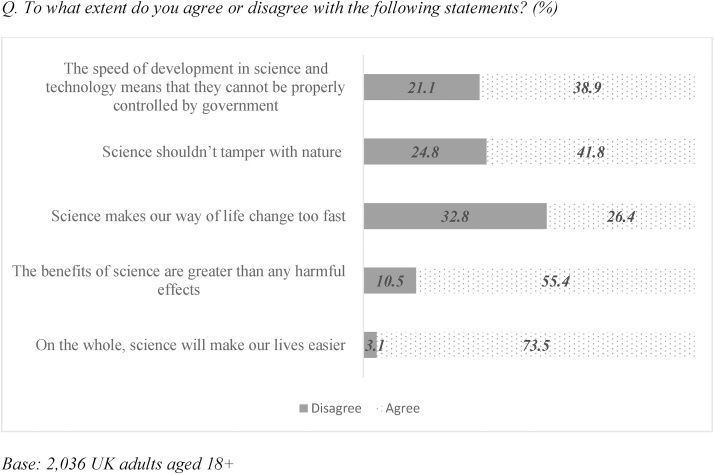
Overall hopes and concerns about science.

In a separate set of questions, we looked at the attitudes to science, scientists and science policy among the UK public. The set of questions used closely matched the set used in PAS (Public Attitude to Science) 2014 survey, conducted by Ipsos MORI on behalf of the UK Department for Business Innovation and Skills (PAS, 2014). Our results agreed with the findings presented in PAS survey report and showed that the UK public continue to see science as beneficial to society. Nearly four-fifths (N = 1495, or 73.5%) of respondents agreed that science will make people’s lives easier, and over half (N = 1128, or 55.4%) thought that the benefits of science outweigh any harmful effects with few disagreeing (N = 214, or 10.5%). On the latter question attitudes differed significantly among age groups (p < 0.001): 64.8% of respondents aged 18–24 agreed with the statement but this dropped to 51.2% in the 55+ age group. Further 38.9% of respondents (N = 793) thought that the speed of development in science and technology means that these areas cannot be properly controlled by government. The 55+ age group were also more likely to agree with the statement that government cannot properly control science (43.1% cf. 38.9% average; p < 0.001) and that science should not tamper with nature (47.4% cf. 41.8% average; p < 0.001) (Fig. 1).

When asked about their level of scientific knowledge about plant science, 52.8% (N = 1075) of respondents felt they were not very well informed, and 23.2% (N = 472) of respondents felt they were not at all informed. Additionally, 54.9% (N = 118) of respondents reported that they were not well informed about scientific research and developments in the area of tree pests and diseases in general, and 24.7% (N = 503) felt they were not at all informed about developments in this area. We found no significant association between respondents’ level of education and level of knowledge. The level of knowledge, however, differed based on respondent’s level of education – 26.4% of those without a university degree reported being not all informed about plant science, versus 21.5% of respondents with a university degree; and 30.2% of respondents without a degree felt not at all informed about scientific research and developments in the area of tree pests and diseases, versus 22.8% of respondents with a degree.

## 4 Discussion and conclusion

Our goal was to provide science and policy with an up-stream ‘steer’ on the likely public acceptability of different tree breeding solutions. The findings from this survey are clear. Whilst there is a firm anti-GM and ‘*we shouldn’t tamper with nature*’ attitude among UK public there is an equally firm and perhaps slightly larger pragmatic attitude that GM (science and technology) should be used if there is a good reason to do so, for example if it can help protect trees from disease and help feed the world. Furthermore, this ‘pragmatic use of science’ attitude is more prevalent among younger generations and urban residents and in the context of urban and plantation rather than countryside settings. Importantly, our survey also picked up a signal of negative attitudes towards options involving non-native trees or stock, which perhaps reflects wider political debate and sentiments regarding the issue of migration in the UK and wider Europe.

The focus group discussions with policy experts and stakeholders revealed a strong, though not unanimous views that a) large investments in finding solutions to ash dieback may not be justified on purely economic grounds and b) letting nature take its course is the only feasible and sensible course of action in natural woodlands (Jepson and Arakelyan, 2016). The survey reported here found that a ‘no action’ option has little public acceptability, which reinforces the same finding from our survey of interested publics (Jepson and Arakelyan, 2017) and similar findings in surveys conducted by the UK’s Forestry Commission in 2013 and 2015 (Forestry Commission, 2015, Forestry Commission, 2013). In short, the UK public expects their government to do something about the problem.

From a scientific and tree-breeding perspective the quickest and most predictable approach is to develop a disease tolerant native ash using genomic techniques, either by accelerated breeding (genomic screening of seeds and seedlings) or Cis-GM (discussions with experts). The former would be the least controversial of the two options; however, a large proportion of the survey population (38% and 37% respectively, Table 7) expressed positive attitude to cis-GM solutions if the tree stock developed was used in urban settings or plantations. Most recently, there has been a significant step forward in developing genetic markers for accelerated breeding – the genome of *F. excelsior* has been sequenced and transcriptomic markers for *Chalara* susceptibility identified (Harper et al., 2016).

An assessment of the cost-effectiveness of accelerated (genomic) breeding vs Cis-GM is beyond the scope of this paper. However, in our view there is a case for investing in both. Developments in the two technologies may interact and over time each may produce varieties of ash suited to different situations, for example *Fraxinus* products of accelerated breeding might be acceptable replacements for ash standards in areas of outstanding natural beauty and Cis-GM ash trees for urban amenity planting and use in timber plantations.

Policy on tree diseases needs to adopt a long-term perspective as the outcomes of policy will only transpire 20–30 years hence. Another clear signal from this study, and also picked up in our survey of ‘interested publics’ (Jepson and Arakelyan, 2017) is that younger and more educated people are more relaxed about the use of genomic techniques to reduce disease susceptibility in native ash trees. Millennials (those who reached adulthood around 2000 or after) have a longer term stake in tree-disease policy, have been less influenced by the GM controversies of the 1990s and appear to hold attitudes more supportive of technical interventions in the makeup of trees. This represents an opportunity for science and policy.

This said, anti-GM attitudes are still prevalent among influential actors in society, and the UK media has a culture of framing science in sensational and emotive terms to generate controversy and attract readers. For example, an established environmental journalist used our invitation to join one of the focus groups to publish an article with the headline ‘With *90% of the UK’s ash trees about to be wiped out, could GM be the answer?*’ (The Observer, 2015). Politicians and policy makers are understandably nervous of negative headlines and a resurfacing of the GM debate. Our findings suggest that the UK government could include genomic solutions in a tree health policy with more confidence if it so wished. It could restate a commitment to evidence-based policy and show that, in the case of trees, there is less public opposition to the use of GM-techniques. This said, the first two survey components revealed that anti-GM attitudes were more prevalent among naturalists who tend to me members of NGOs with significant policy lobbies and also among tree policy experts. A clear view from the focus groups was that reducing disease susceptibility using GM techniques should be used as a last resort option.

In this survey, of particular significance is the finding that 40% or respondents held the view that humans should not interfere with nature, which is significantly correlated with respondents’ anti-GM attitudes. In a study of media, web and NGO articles on the purpose of conservation, Ladle and Gillson (2009) found that the ‘balance of nature metaphor’ remains prominent and this may explain the preponderance of the ‘noninterference with nature’ attitude among UK publics. This attitude is however out of step with much current scientific thinking and practice. Three decades ago, there was a conceptual shift within the environmental and social science from an emphasis on ecosystem stability and balance to embracing the reality of flux and change. Further, the concept of nature as something pure and separate from humanity has been dismantled by social theorists and it is increasingly recognized that there are multiple natures with multiple degrees of human influence and interaction. Further, modification of once wild species to serve humans is central to history, culture and society.

The finding that the attitude that it is wrong to “meddle” or “tamper” with nature is so prevalent among UK publics suggests a failure of public education. This is not in any way to suggest that GM techniques are risk free and, indeed, there is a significant amount of uncertainty and limited scientific evidence to support the use of GM-techniques in fighting tree diseases. Rather, it is to make the point that if publics believe that there is a balance of nature that can be unsettled they will be less able to a) assess the merit of existing (and increasing) scientific evidence relating to GM technologies and b) be less prepared to deal with environmental change, including the spread of tree diseases which seems inevitable. This suggests that the development of an effective tree disease policy needs to go hand-in-hand with a broader strategy of public education involving other policy areas dealing with the impacts of accelerated environmental change.

Finally, this study enables comparison of the cost-effectiveness of two surveys: a survey of interested public using a bespoke sampling frame that cost approximately £10 000 and 6 academic man-months to design, implement and analyse, and survey of the general public using a commercial sampling frame that cost £5000 and 3 academic man months to deliver. The second more cost-efficient, and also more comprehensive survey, produced very similar findings to the first and added additional information on underlying world views. It also had the added advantage that respondents could be expected to commit more time to completing the survey. The more costly bespoke survey, however, generated data on more policy-empowered publics and its design prompted ideas that may not have been incorporated in the general survey (e.g. the importance of hobbies). However, the resolution of the bespoke survey could be replicated with the development of standardized questions on interest group membership and nature-related pass times, such that public attitude data to contribute and steer policy and science decisions could be generated relatively quickly and at reasonable costs.
